# The *Arabidopsis* MYB5 transcription factor interacts with CASEIN KINASE2 BETA3 subunit in a yeast two-hybrid system

**DOI:** 10.17912/micropub.biology.000486

**Published:** 2021-10-07

**Authors:** Ross S Napoli, Patrick J Allen, Roger W Parish, Song Feng Li

**Affiliations:** 1 Department of Animal, Plant and Soil Sciences, AgriBio, Centre for AgriBiosciences, La Trobe University, Bundoora, Melbourne, Victoria 3086, Australia.; 2 School of Biosciences, University of Melbourne, Parkville, Victoria 3010, Australia.

## Abstract

*Arabidopsis thaliana* MYB5 collaborates with TRANSPARENT TESTA GLABRA1 (TTG1) and basic-Helix-Loop-Helix (bHLH) transcription factors to regulate seed coat, trichome and root cell differentiation. Using a yeast two-hybrid system we show that the N-terminal region of MYB5 binds directly to the serine/threonine CASEIN KINASE2 BETA3 (CK2β3) subunit. Functions of the CASEIN KINASE2 (CK2) complex include facilitating phosphorylation of MYB transcription factors and cell cycle checkpoint regulatory proteins. Purified recombinant MYB5 protein was found to bind only weakly *in vitro* to the promoter of *ALPHA/BETA ESTERASE/HYDROLASE4 (ABE4)*, a known MYB5 target gene. We propose that phosphorylation of MYB5 facilitated by the MYB5-CK2β3 interaction enhances MYB5 binding to its target gene promoters.

**Figure 1. Yeast two-hybrid analysis of MYB5 interaction with CK2β3 protein kinase subunit and EMSA binding of MYB5 to the ABE4 promoter:**
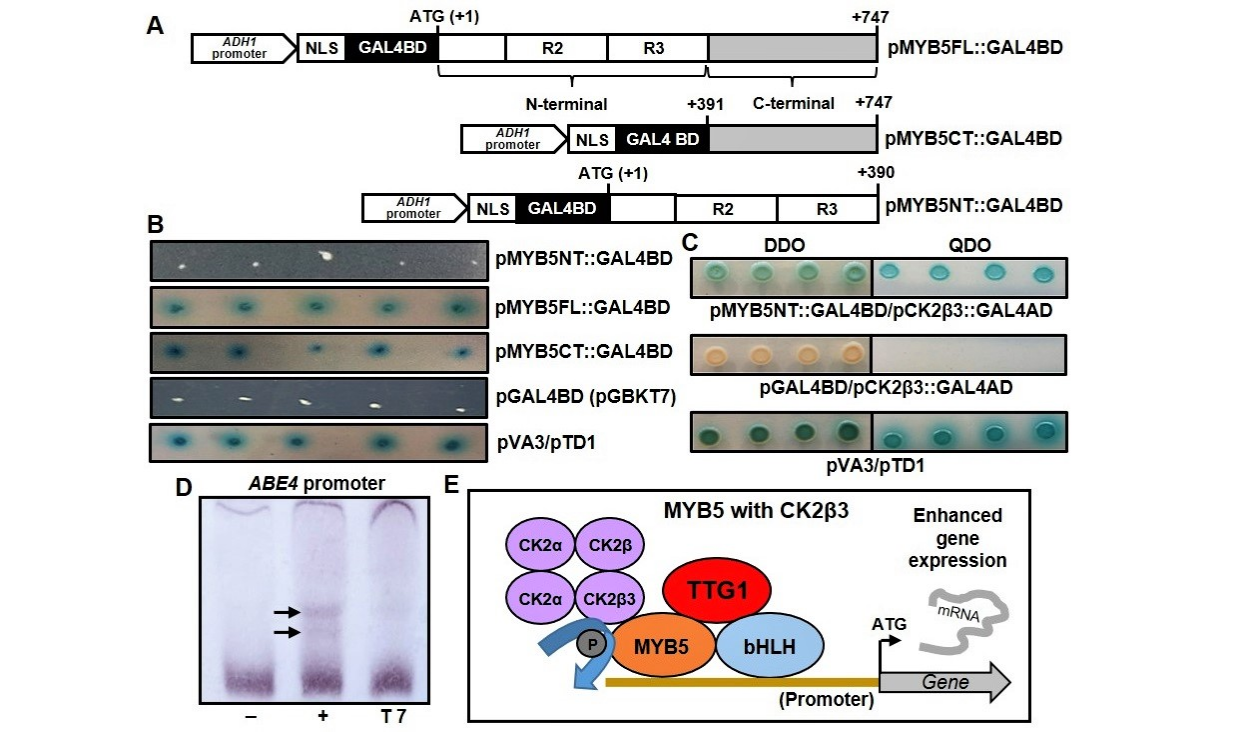
**(A)** Schematic diagram showing three candidate MYB5 bait constructs containing the cDNA nucleotide sequences coding for MYB5 full length, N-terminal and C-terminal regions. The three *MYB5* cDNA sequences were each fused in frame with *GAL4BD* in pGBKT7 plasmids. *ADH1: ALCOHOL DEHYDROGENASE1*, ATG: Methionine start codon, NLS: Nuclear localisation sequence, R2: MYB repeat 2, R3: MYB repeat 3. **(B)** Auto-activation screening of the candidate bait constructs. pMYB5FL::GAL4BD and pMYB5CT::GAL4BD colonies grew and turned blue while pMYB5NT::GAL4BD colonies failed to do so (no auto-activation). **(C)** Confirmation of protein-protein interaction between MYB5 and CK2β3 in yeast. Colonies transformed with pMYB5NT::GAL4BD and pCK2β3::GAL4AD grew while colonies transformed with pGAL4BD (empty pGBKT7 vector) and pCK2β3::GAL4AD failed to grow. Positive control: pVA3 (VA3 protein) and pTD1 (TD1 protein). **(D)** Electrophoretic mobility shift assay (EMSA) was used to determine MYB5 protein binding to a target promoter *in vitro*. The EMSA analysis showed weak binding affinity of AtMYB5trunc protein to an *ABE4 (At2g23580)* promoter sequence containing two MYB-related regulatory elements (MYBCORE and MYBLEPR binding motifs). Two weak band shifts were detected within the same probe indicating that MYB5 binds weakly to the enriched region containing the MYBCORE (CNGTTR) and MYBLEPR (GTTAGTT) binding motifs (Li **et al.*,* 2020). Arrows indicate probe shift, – indicates labelled probe only, + indicates labelled probe plus AtMYB5 protein, T7 indicates labelled probe with the addition of T7 antibody. **(E)** Model for MYB5-CK2β3 interaction and MYB5 protein-DNA binding for activation of MYB5 target gene expression in plants. CK2 may facilitate phosphorylation of MYB5 directly or by recruiting additional kinase enzymes thereby assisting with MYB5-DNA binding to target gene promoters. bHLH: basic-Helix-Loop-Helix, (P): Phosphate group.

## Description

The Arabidopsis *(Arabidopsis thaliana)* MYB5 (At3g13540) transcription factor (TF) forms transcriptional regulatory (MBW) complexes with basic-Helix-Loop-Helix (bHLH) and TRANSPARENT TESTA GLABRA1 (TTG1) (WD-repeat) TF proteins. These MBW complexes regulate seed coat, trichome and root cell differentiation via a multi-tiered transcriptional mechanism (Golz **et al.*,* 2018; Li **et al.*,* 2020). MYB5 is partially redundant with MYB23 and TRANSPARENT TESTA2 (TT2) (another MYB protein, MYB123) in MBW complex formation. TTG1 is subject to site-specific phosphorylation by SHAGGY-LIKE KINASE11 (SK11) and SK12 which inhibits TTG1 interactions with TT2 (Li **et al.*,* 2018). MYB5 also possesses potential phosphorylation sites at the N-terminus (Li **et al.*,* 1996).

To identify proteins interacting with MYB5, the Matchmaker® Gold Yeast Two-hybrid system was used to screen a prey cDNA library. Three candidate bait constructs contained cDNA nucleotide sequences encoding the MYB5 full length, C-terminal and N-terminal regions (Fig 1A), respectively. The constructs containing full length MYB5 and the MYB5 C-terminal region activated *MEL1* and *AUR1-C* reporter genes resulting in blue colonies (Fig 1B) indicating that the C-terminal region acts as an activator motif in yeast. The MYB5 N-terminal construct (pMYB5NT::GAL4BD) failed to activate the reporter genes (Fig 1B) and was therefore used as the bait for library screening. On the screening plates, five yeast colonies were identified and their prey cDNA fragments sequenced. Three of the five cDNA sequences encoded a serine/threonine protein kinase *CAESIN KINASE2 BETA3 (CK2β3) (At3g60250)* subunit fused in frame with the GAL4AD domain-encoding nucleotide sequence. The remaining two cDNA sequences encoded *Hypothetical/HEAT RESPONSE PROTEIN (At5g10010)* and *PHOSPHOGLUCOSE ISOMERASE (At4g24620)*. The dependency of pCK2β3::GAL4AD on pMYB5NT::GAL4BD in activating four reporter genes, namely *AUR1-C*, *HIS3, ADE2* and *MEL1,* was then confirmed (Fig 1C).

MYB5 directly regulates *ALPHA/BETA ESTERASE/HYDROLASE1 (ABE1, At2g23550)* and *ABE4 (At2g23580)* expression (Li **et al.*,* 2020). The electrophoretic mobility shift assay (EMSA) was used to determine *in vitro* binding of purified MYB5 protein to the *ABE4* promoter. A truncated MYB5 protein containing the MYB DNA-binding domain was expressed in *E. coli* and purified. The *ABE4* promoter probe (Suppl. Fig 1) contains two sequences identified as MYB5 binding sites using chromatin immunoprecipitation (Li **et al.*,* 2020). The EMSA identified two weak band shifts (Fig 1D) indicating that MYB5 binds weakly to the *ABE4* promoter region *in vitro* and may therefore be subject to phosphorylation/activation for enhanced MYB-DNAbinding *in vivo*.

The Arabidopsis CK2β3 subunit is part of the CK2 holoenzyme which is a tetramer consisting of two regulatory β-subunits and two catalytic α-subunits collectively implicated in a number of developmental and stress-responsive pathways (Mulekar and Huq, 2013; Wei **et al.*,* 2021). Known CK2 substrates primarily include transcription factors or regulatory proteins (Mulekar and Huq, 2013). *CK2β3* is highly expressed in developing seeds and has an expression pattern overlapping that of *MYB5, TTG1, ABE1* and *ABE4* (Winter **et al.*,* 2007). CK2β3localises to the nucleus and cytosol (Salinas **et al.*,* 2006) and can modulate the activity of the MYB-related CIRCADIAN CLOCK-ASSOCIATED1 (CCA1) both by direct physical interaction *in vivo* and phosphorylation (Sugano **et al.*,* 1999). CK2β3 shares 75% amino acid sequence similarity with CK2β1 and 71% with CK2β2 (Sugano **et al.*,* 1998). CK2β3 and CK2β4 subunits share 87% amino acid sequence identity and 92% sequence similarity. Moreover, like *CK2β3*, *CK2β4* has also been linked to the phosphorylation of CCA1 as over-expression of both genes leads to period-shortening of circadian clock-related gene expression (Perales **et al.*,* 2006). Although CK2β3 does not exhibit phosphorylation activity, it has been shown to facilitate CCA1 binding to the promoter of target gene *CHLOROPHYLL A/B BINDING PROTEIN1 (CAB1)* (Sugano **et al.*,* 1998). CK2 also interacts with the MYB-related LATE ELONGATED HYPOCOTYL (LHY) and YING YANG1 (YY1) via CK2β3 and phosphorylates LHY and YY1 proteins *in vitro* (Sugano **et al.*,* 1999; Wu and Li, 2017).

The transcriptional activities of *Pinus taeda* MYB4 (PtMYB4) and PtMYB46 are positively regulated by phosphorylation (Morse **et al.*,* 2009) although phosphorylation of TFs such as LTF1 (which regulates lignin biosynthesis in *Populus*) can result in its degradation (Gui **et al.*,* 2019). In contrast, DNA binding of Arabidopsis MYB15, a transcriptional repressor of cold signalling, is reduced by phosphorylation (Kim **et al.*,* 2017). While specific MYB5-CK2β3 interactions *in planta* are yet to be determined, CK2β3 may facilitate direct MYB5 binding to its target gene promoters or may phosphorylate MYB5 indirectly by recruiting the CK2 catalytic α-subunits. The MYB5 N-terminal region contains several threonine and serine residues which are potential phosphorylation sites (Li **et al.*,* 1996) and phosphorylation of these residues may enhance MYB5 protein-DNA binding affinity. Alternatively, as MYB5-TTG1 and/or MYB5-bHLH interactions were not detected in the initial yeast two-hybrid screening, MYB5 may also require activation to facilitate TF protein-protein binding. TTG1 is subject to site-specific phosphorylation which regulates binding with TT2 (MYB123) to form some MBW complex combinations (Li **et al.*,* 2018). MYB5-CK2B3 interactions and proposed phosphorylation may contribute to MYB5-bHLH-TTG1 (MBW) complex formation which, in turn, may further increase MYB5 protein-DNA binding affinity *in vivo*.

In the proposed model (Fig 1E), MYB5-bHLH-TTG1 complexes activate the expression of target genes by binding to their promoters. CK2β3 interacts with MYB5 transiently and recruits the CK2 holoenzyme to phosphorylate MYB5 (Fig 1E) resulting in more active MYB5-bHLH-TTG1 complexes thereby enhancing expression of MYB5 target genes.

## Methods

**Plasmid construction**

For yeast two-hybrid (Y2H) analysis, MYB5 full length, C-terminal and N-terminal nucleotide sequences were PCR amplified from cDNA prepared from developing wild-type (Col) siliques using Y2HFULLF/Y2FULLR [pMYB5FL], Y2HFULLF/Y2HNTR [pMYB5CT] and Y2HCTF/Y2FULLR [pMYB5NT] primer combinations, respectively. The MYB5 cDNA sequences were ligated into the pGBKT7 yeast expression vector at the EcoR1 and BamH1 restriction sites, generating pMYB5FL::GAL4BD, pMYB5CT::GAL4BD and pMYB5NT::GAL4BD vectors, respectively. For EMSA analysis, a truncated MYB5-encoding cDNA sequence (410 nucleotides) containing the MYB DNA-binding domain sequence with the truncation after the GIDPOTHK polyadenylation site was PCR amplified from wild-type (Col) seed cDNA using MYBPROTF and MYBTRUNCR primers. Truncation of MYB proteins beyond the MYB domain does not appear to affect binding specificity (Li and Parish, 1995; Phan **et al.*,* 2011). The truncated MYB5 fragment was ligated into the pRSETA expression vector (Invitrogen) at Xho1 and EcoR1 restriction sites and was fused to a 6X polyhistidine tag and a T7 leader peptide coding sequence and driven by the T7 promoter which was PCR amplified using T7F and PGADT7R primers.

**Yeast Two-Hybrid analysis**

The yeast two-hybrid (Y2H) assay was performed using the Matchmaker™ Gold Yeast Two-Hybrid system (Clontech) and all protocols were adapted from the Clontech Yeast Protocols Handbook and the Matchmaker™ Gold Yeast Two-Hybrid user manual. To determine the autoactivation of candidate bait constructs, Y2HGold cells were transformed with the three constructs and inoculated onto dropout (-trp) media containing x-α-gal and auribasidin A. Five technical replicates are presented (Fig 1B). pGAL4BD (empty pGBKT7 vector) was used as a negative control and pVA3/pTD1 co-transformed cells were used as a positive control. The Mate & Plate™ Library – Universal Arabidopsis (Normalised) (Clontech) was used which consists of a cDNA library cloned into the GAL4AD vector (pGADT7, prey) and transformed into yeast strain Y187. Y2HGold yeast cells transformed with pMYB5NT::GAL4BD (bait) were mated with the Y187 cells expressing the Mate & Plate™ Library made from 11 Arabidopsis tissues. The mated cultures were plated onto the double dropout (–trp –leu) media (DDO) containing x-α-gal and auribasidin A. to identify bait and prey interaction. Yeast cells were then inoculated on double-dropout (DDO) medium (-leu/-trip) plus x-α-gal and quadruple-dropout (QDO) (-ade/-his/-leu/-trip) medium plus x-α-gal and auribasidin A. Double-dropout medium (-leu/-trp) selects for the presence of both pGADT7 and pGBKT7. Figure 1C depicts one representative biological replicate of MYB5-CK2β3 binding. Four technical replicates are presented. In total, 3 out of 5 (60%) of the positive colonies from initial Y2H screening were confirmed as positive MYB5-CK2β3 interactions.

**Electrophoretic Mobility Shift Assay (EMSA)**

Recombinant MYB5 protein expressed in *E. coli* was purified using Ni-NTA Superflow columns (Qiagen) according to the manufacturer’s protocol. EMSA was performed using a non-radioactive protocol (adapted from Phan **et al.*,* 2011) and the protocol described by Li and Parish (1995). The ABE4MCOREF and ABE4MCORER primers were designed to PCR amplify a probe of 150 to 200 nucleotides in length based on the promoter region enriched in ChIP analysis (Li **et al.*,* 2020). Protein-probe binding was performed using 400 ng total protein, 3 mL of binding buffer (20 mM NaCl, 5 mM MgCl_2_, 20 mM Tris [pH 8.0], 10% glycerol, 0.5 mM EDTA and 0.5 mM DTT) with 1 mL of poly d(I-C). The truncated MYB5 protein was expressed in *E. coli* and purified. SDS-PAGE and Coomassie Blue staining detected bands of approximately ~21kDa molecular weight in two independent replicates. Western Blot analysis using an anti-T7 epitope tag antibody detected ~21k kDA bands in each lane corresponding to the truncated MYB5 protein replicates. EMSA probes were labelled with digoxigenin via PCR using digoxigenin-labelled dUTP (Roche).

## Reagents

**Primer oligonucleotide sequences**

**Table:** 

Primer name	Primer sequence (5′ to 3′ orientation)
Y2HFULLF	GAGAGAGAATTCATGATGTCATGTGGTGGG
Y2FULLR	GAGAGAGGATCCCTAGTCATGTCCTAAGCTAGAAGA
Y2HCTF	AGAGAGAATTCGGAATTGATCCTCAAACCCACAAG
Y2HNTR	GAGAGAGGATCCTTGCCTTAAAAGTTTCTTACGAAG
T7F	TAATACGACTCACTATAGGGC
PGADT7R	AGATGGTGCACGATGCACAG
MYB5PROTF	CGAGCTCGAGATGATGTCATGTGGTGGGAAGAAGCC
MYB5PROTR	GAGAGAATTCCTAGTCATGTCCTAAGCTAGAAGA
MYB5TRUNCR	TCTCGAATTCCTACTTGTGGGTTTGAGGATCAATTCC
ABE4MCOREF	GGTTCAGAATTTATTACTTACTTTGGTTG
ABE4MCORER	CGATGGTCACTTTCCTCATACTCTTTC

## References

[R1] Golz JF, Allen PJ, Li SF, Parish RW, Jayawardana NU, Bacic A, Doblin MS (2018). Layers of regulation - Insights into the role of transcription factors controlling mucilage production in the Arabidopsis seed coat.. Plant Sci.

[R2] Gui J, Luo L, Zhong Y, Sun J, Umezawa T, Li L (2019). Phosphorylation of LTF1, an MYB Transcription Factor in Populus, Acts as a Sensory Switch Regulating Lignin Biosynthesis in Wood Cells.. Mol Plant.

[R3] Kim SH, Kim HS, Bahk S, An J, Yoo Y, Kim JY, Chung WS (2017). Phosphorylation of the transcriptional repressor MYB15 by mitogen-activated protein kinase 6 is required for freezing tolerance in Arabidopsis.. Nucleic Acids Res.

[R4] Bernstein DT, Sochacki KR, Jafarnia KK (1970). Sterile Abscess with Subsequent Iatrogenic Draining Sinus Tract Formation 3 Years After FiberWire and ENDOBUTTON Distal Biceps Brachii Tendon Repair: A Case Report.. JBJS Case Connect.

[R5] Li SF, Allen PJ, Napoli RS, Browne RG, Pham H, Parish RW (2020). MYB-bHLH-TTG1 Regulates Arabidopsis Seed Coat Biosynthesis Pathways Directly and Indirectly via Multiple Tiers of Transcription Factors.. Plant Cell Physiol.

[R6] Li SF, Milliken ON, Pham H, Seyit R, Napoli R, Preston J, Koltunow AM, Parish RW (2009). The Arabidopsis MYB5 transcription factor regulates mucilage synthesis, seed coat development, and trichome morphogenesis.. Plant Cell.

[R7] Li SF, Santini JM, Nicolaou O, Parish RW (1996). A novel myb-related gene from Arabidopsis thaliana.. FEBS Lett.

[R8] Li SF, Parish RW (1995). Isolation of two novel myb-like genes from Arabidopsis and studies on the DNA-binding properties of their products.. Plant J.

[R9] Morse AM, Whetten RW, Dubos C, Campbell MM (2009). Post-translational modification of an R2R3-MYB transcription factor by a MAP Kinase during xylem development.. New Phytol.

[R10] Mulekar JJ, Huq E (2013). Expanding roles of protein kinase CK2 in regulating plant growth and development.. J Exp Bot.

[R11] Perales M, Portolés S, Más P (2006). The proteasome-dependent degradation of CKB4 is regulated by the Arabidopsis biological clock.. Plant J.

[R12] Phan HA, Iacuone S, Li SF, Parish RW (2011). The MYB80 transcription factor is required for pollen development and the regulation of tapetal programmed cell death in Arabidopsis thaliana.. Plant Cell.

[R13] Salinas P, Fuentes D, Vidal E, Jordana X, Echeverria M, Holuigue L (2006). An extensive survey of CK2 alpha and beta subunits in Arabidopsis: multiple isoforms exhibit differential subcellular localization.. Plant Cell Physiol.

[R14] Sugano S, Andronis C, Ong MS, Green RM, Tobin EM (1999). The protein kinase CK2 is involved in regulation of circadian rhythms in Arabidopsis.. Proc Natl Acad Sci U S A.

[R15] Sugano S, Andronis C, Green RM, Wang ZY, Tobin EM (1998). Protein kinase CK2 interacts with and phosphorylates the Arabidopsis circadian clock-associated 1 protein.. Proc Natl Acad Sci U S A.

[R16] Wei P, Demulder M, David P, Eekhout T, Yoshiyama KO, Nguyen L, Vercauteren I, Eeckhout D, Galle M, De Jaeger G, Larsen P, Audenaert D, Desnos T, Nussaume L, Loris R, De Veylder L (2021). Arabidopsis casein kinase 2 triggers stem cell exhaustion under Al toxicity and phosphate deficiency through activating the DNA damage response pathway.. Plant Cell.

[R17] Winter D, Vinegar B, Nahal H, Ammar R, Wilson GV, Provart NJ (2007). An "Electronic Fluorescent Pictograph" browser for exploring and analyzing large-scale biological data sets.. PLoS One.

[R18] Wu XY, Li T (2017). A Casein Kinase II Phosphorylation Site in AtYY1 Affects Its Activity, Stability, and Function in the ABA Response.. Front Plant Sci.

